# A DNA Microarray-Based Assay to Detect Dual Infection with Two Dengue Virus Serotypes

**DOI:** 10.3390/s140507580

**Published:** 2014-04-25

**Authors:** Alvaro Díaz-Badillo, María de Lourdes Muñoz, Gerardo Perez-Ramirez, Victor Altuzar, Juan Burgueño, Julio G. Mendoza-Alvarez, Jorge P. Martínez-Muñoz, Alejandro Cisneros, Joel Navarrete-Espinosa, Feliciano Sanchez-Sinencio

**Affiliations:** 1 Department of Genetics and Molecular Biology, Centro de Investigación y de Estudios Avanzados del IPN (CINVESTAV-IPN), Mexico D.F., 07360, Mexico; E-Mails: alvaro@cinvestav.mx (A.D.-B.); gperezr@cinvestav.mx (G.P.-R.); 2 Coordinación Académica, Universidad Autónoma de la Ciudad de México, México D.F., 09620, México; 3 Centro de Investigación en Micro y Nanotecnología (MICRONA), Universidad Veracruzana, Boca del Rio, Veracruz, 94294, Mexico; E-Mail: valtuzar@gmail.com; 4 Centro Internacional de Mejoramiento de Maíz y Trigo (CIMMYT), Texcoco, Estado de Mexico, 56237, Mexico; E-Mail: jabfpc@yahoo.com; 5 Department of Physics, Centro de Investigación y de Estudios Avanzados del IPN (CINVESTAV-IPN), Mexico D.F., 07360, Mexico; E-Mails: juliogma1@gmail.com (J.G.M.-A.); fsanchez@fis.cinvestav.mx (F.S.-S.); 6 Laboratorio Estatal de Salud Pública de Oaxaca, Servicios de Salud de Oaxaca, Oaxaca, 71257, Mexico; E-Mail: monterrey2bios@hotmail.com; 7 Escuela de Medicina Veterinaria y Zootecnia, Universidad Autónoma Benito Juarez de Oaxaca, 68120, Oaxaca, Mexico; E-Mail: alecis@msn.com; 8 División de Epidemiologia, Coordinación de Programas Integrados de Salud, Instituto Mexicano del Seguro Social, Mexico D.F., 06700, Mexico; E-Mail: joel.navarrete@imss.gob.mx

**Keywords:** dengue virus, humans, *Aedes*, microarrays

## Abstract

Here; we have described and tested a microarray based-method for the screening of dengue virus (DENV) serotypes. This DNA microarray assay is specific and sensitive and can detect dual infections with two dengue virus serotypes and single-serotype infections. Other methodologies may underestimate samples containing more than one serotype. This technology can be used to discriminate between the four DENV serotypes. Single-stranded DNA targets were covalently attached to glass slides and hybridised with specific labelled probes. DENV isolates and dengue samples were used to evaluate microarray performance. Our results demonstrate that the probes hybridized specifically to DENV serotypes; with no detection of unspecific signals. This finding provides evidence that specific probes can effectively identify single and double infections in DENV samples.

## 1. Introduction

Dengue is a mosquito-borne viral infection and a major global public health problem [1]. The most common infection produces the classical dengue fever (DF), which is characterized by a sudden onset of rash, high fever, headache, and backache. The main clinical manifestations, namely dengue haemorrhagic fever (DHF) and dengue shock syndrome (DSS), are responsible for high morbidity and mortality rates every year. Over 40% (2.5 billion) of the population in 100 tropical and subtropical countries continue to live under the threat of contracting dengue infection. It is estimated that 100 million cases of DF, 500,000 cases of DHF, and 25,000 deaths are reported annually worldwide [1]. The fatality rate due to DHF may be reduced significantly with the aid of modern supportive therapies based on early diagnosis of the specific viral infection, but in most cases, these deaths are due to the lack of an early diagnosis of DENV infection, which is caused by four distinct serotypes: DENV-1, DENV-2, DENV-3, and DENV-4. Despite extensive research, there are currently no vaccines available [2,3], although candidate dengue vaccines have recently entered phase III trials in Asia and Latin America [4,5].

DENV (genus *Flavivirus*, family *Flaviviridae*) is mosquito-borne and mainly transmitted by *Aedes* (*Stegomyia*) *aegypti* (L.) and *Aedes* (*Stegomyia*) *albopictus* (*Skuse*) (*Diptera*: *Culicidae*), which are infected through receptors in the midgut [6–8]. This virus has a positive-strand RNA genome of approximately 11,000 bases, which encodes three structural and seven non-structural proteins. The detection and typing of this virus, not only in the patient but also in the vector, will lead to the development of new strategies for controlling and handling DENV outbreaks. In addition, surveillance of mosquitoes infected with DENV can help monitor infection rates within vector populations harbouring specific serotypes and provide early warning of pending epidemics.

There is a growing need for rapid and reliable methods for serotype identification of DENV infections in human serum specimens and mosquito vectors. The identification of DENV serotypes is of particular importance because these infections have been associated with DHF/DSS [9–11]. Furthermore, DENV testing is required to confirm the diagnosis of DENV infection in order to differentiate it from other febrile tropical illnesses. Conventional diagnosis of DENV infections includes the detection of virus in serum by isolation in culture and detection of specific viral molecules, such as genome RNA or dengue antigens [12,13], or by detection of specific anti-dengue antibodies [14]. Isolation of DENV provides the most direct and conclusive approach, although virus isolation takes 8 to 10 days, depending on titre. Anti-dengue antibodies can be detected within the first 5 days of symptoms or the 4 to 5 days required for the immune system to produce a sufficient amount of antibodies. Moreover, antibodies produce misleading results in secondary infections due to cross-reactivities among serotype-specific antibodies and other flavivirus antibodies. Reverse transcription-polymerase chain reaction (RT-PCR) is another potential method for detection of viral RNA [15–19]. Initial studies demonstrated the need to obtain virus isolates from C6/36 cells infected with each virus sample before detection by RT-PCR [20,21]. This method is also limited by the number of species that can be detected and identified in a single test, often requiring multiple parallel reactions [22]. Real-time RT-PCR is more sensitive than RT-PCR [23] and may be used to develop a diagnostic test. Single-tube or parallel multiplex PCR assays are alternative methods that can be coupled to hybridisation using conventional microarrays or flow-thru DNA chips [22,24]. Previous studies have described the use of DNA microarrays to study respiratory viruses in hospitalised infants admitted to intensive care units as well as in paediatric units and in adult patients with influenza-like illnesses [25–27]. These authors found that global detection rates appeared to be higher with DNA microarrays than with conventional virological methods such as direct immunofluorescence, virus isolation, and RT-PCR. This is the case not only in suspected human cases but also in mosquito populations [16]. Recently, several researchers have reported the use of fully automated real-time RT-PCR assays for the detection and serotype identification of DENV in patient sera [28,29]. However, this method does not allow multiplexing for more than four targets. In contrast, microarrays are routinely used in fields where a multiplex detection approach is required [22,24,30]. Furthermore, DNA microarray technologies have found applications in disease diagnosis [30–32], gene discovery [33], genetic studies [34], pharmacogenomics [35], and toxicology [36].

Here, we demonstrate the application of this DNA microarray technology to detecting single or dual infection with two dengue virus serotypes. The system is capable of detecting DENV serotype (DENV-1, DENV-2, DENV-3, and DENV-4) during the early phase of illness or in mosquito populations. The technique provides sufficient sensitivity and reproducibility for the diagnosis of dengue in human and mosquito samples. Thus, our biosensor will facilitate early dengue diagnoses and provide a reliable estimation of dengue infection in mosquito vectors for prevention, surveillance, and control programs.

## 2. Experimental Section

### 2.1. Materials

All chemicals and solvents were purchased from Fluka-Sigma (St. Louis, MO, USA) at the highest analytical grades. Corning UltraGAPS, Aminosilane Coated Slides (Corning Incorporated, Corning, NY, USA), or Nexterion^®^ Slide A+Aminosilane-Coated Substrate (Schott Nexterion, Jena, Germany) were used in all experiments.

### 2.2. Viruses

The flaviviruses used in this study included DENV-1, strain Hawaii (HA1-1944); DENV-2, strain New Guinea C (NGC-1944); DENV-3, strain Philippines (H-87); and DENV-4, strain Philippines (H-241-1946). The viruses were obtained from Dr. Duane J. Gubler (Division of Vector-borne Infectious Diseases, Centers for Disease Control, Fort Collins, CO, USA).

### 2.3. Serum Samples

Serum specimens were collected from 197 patients in 2000, 2001, and 2004–2006 in an epidemiologic surveillance program carried out by the Secretaria de Salud de Oaxaca and the Instituto Mexicano del Seguro Social (IMSS) of Veracruz, Mexico. DENV was evaluated by Mac-ELISA (IgM Antibody Capture Enzyme-Linked Immunosorbent Assay, MAC-ELISA; PANBIO, Brisbane, Australia) at the Secretaria de Salud [37]. This study used excess samples that remained after diagnostic testing and were stored at −70 °C. The study was reviewed and approved by the Institutional Review Board of the IMSS (Commission of Scientific Research) and the Bioethical Commission for Research in Humans of Centro de Investigación y de Estudios Avanzados del IPN (Comité de Bioética Para la Investigación en Seres Humanos, COBISH-CINVESTAV).

### 2.4. Mosquito Collections

Mosquitoes were collected from at least four sites in the Mexican states of Quintana Roo, Yucatan, Campeche, Tabasco, Chiapas, Veracruz, Oaxaca, Puebla, Guerrero, Morelos, Hidalgo, Mexico, Tamaulipas, Queretaro, Guanajuato, Michoacán, Colima, Jalisco, and Nayarit. Geographic locations and numbers of adult mosquitoes collected at natural sites in each city are listed in Table 1. Pools of ten adult mosquitoes were examined at each site to confirm their identities as *Ae. aegypti* or *Ae. albopictus* and then stored at −70 °C.

### 2.5. Isolation of Dengue Virus

*Aedes albopictus* clone C6/36 cells were grown at 28 °C as described [38] to extract viral RNA from strains maintained in the laboratory and used as positive controls in our assays. After 18 h culture, the cells (2 × 10^6^ per 100-mm plate) were infected with 0.2 mL DENV-1, -2, -3 or -4 with an input MOI of 600 PFU per plate and incubated at 28 °C for 10 days.

### 2.6. RNA Extraction

Total RNA was isolated from cell culture supernatants containing viruses obtained from infected cells and from mosquitoes collected and frozen in the field. RNA was obtained using Trizol LS (GIBCO BRL, Gaithersburg, MD, USA) according to the manufacturer's recommendations. Total RNA was also obtained from acute-phase plasma collected from patients in the epidemiologic surveillance program (Secretaria de Salud de Oaxaca, Mexico from 2000, 2001 and 2004–2006) using the Viral Nucleic Acid Extraction kit from Real Genomics (Real Biotech Corporation, Banqqiao, Tapei, Tiwan). The RNA was suspended in 50 μL H_2_O treated with diethylpyrocarbonate (DEPC, Sigma-Aldrich) and used as a template for reverse transcription-polymerase chain reaction (RT-PCR).

### 2.7. RT-PCR Assay

One-step RT-PCR (SuperScript One-Step RT-PCR with Platinum Taq, Invitrogen, Carlsbad, CA, USA) was performed to identify the optimal primers for DENV serotype detection. Primers targeting the C-prM [18], NS1 [17], NS3 [19,39], and NS5 [15] genes yielded products of different sizes (Table 2). Extracted RNA (5 μL) was used as a template in a 25-μL reaction volume. Assays used primers specific for DENV-1–4, but not Japanese encephalitis, Kunjin, or yellow fever viruses [19], and consensus primers common to several flaviviruses (Japanese encephalitis, Kunjin or yellow fever), including dengue viruses. Cycling conditions have been reported previously for each primer set (Table 2) [17–19,39]. Reaction mixtures were stored at −20 °C until further processing.

### 2.8. Probe Labelling

DENV-1, DENV-2, DENV-3, and DENV-4 specific primers (Table 2) were synthesized with the 5′ C6 amino linker by The Midland Certified Reagent Company (Midland, TX, USA). Each pair of oligonucleotides was validated by RT-PCR to verify amplification of products of the expected size.

### 2.9. DNA Microarray Fabrication

**Figure 1.**
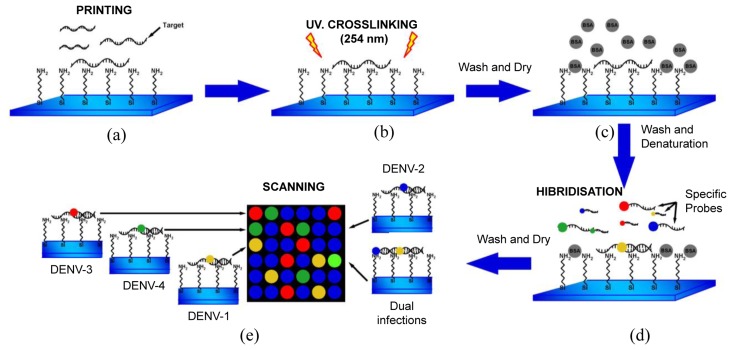
(**a**) DNA microarray targets were printed on NEXTERION^®^ Slide A+ aminosilane coated slides. (**b**) DNA was UV-cross linked at 250 mJ and slides were dried for 20 min at 75 °C. (**c**) Microarrays were blocked with pre-hybridisation buffer (4× SSC, 1% SDS, and 10 mg·mL^−1^ BSA) for 45 min at 42 °C. (**d**) Specific fluorescent probes (Table 2) for DENV-1, -2, -3, or -4 (5 μL) were hybridised to the DNA microarray. (**e**) After washing, the slides were scanned to detect Texas Red (583 nm), FAM (494 nm), Cy5 (530 nm), and Yakima yellow (646 nm).

DNA microarray targets (RT-PCR product of DV1 and DV3) [19] were immobilized as follows (Figure 1). Targets (50–100 ng·μL^−1^) were denatured (10 min at 92 °C) and diluted in buffer [1.6 mol·mL^−1^ CHAPS, 1% CAPS, 50% DMSO buffer (pH 6.5)], and then printed on NEXTERION® Slide A+ aminosilane-coated slides with a Robot Spotter GeneTAC G3 (Genomic Solutions, Harvard Bioscience, Boston, MA, USA) at 55% relative humidity and a temperature of 20 °C to 25 °C (Figure 1a). Each solid pin delivered a volume of approximately 1 nL, which was designed to create a spot diameter of 300 μm. The centre-to-centre spacing of bright spots was 500 μm. The DNA was UV (254 nm)-cross linked at 250 mJ cm^−2^ and the slides were incubated for 20 min at 75 °C as shown in Figure 1b, followed by extensive washing with 0.1% SDS and two rinses with double-deionized water. The chips were blocked with pre-hybridisation buffer (4× SSC, 1% SDS, and 10 mg·mL^−1^ BSA) for 45 min at 42 °C, washed with double-deionized water for 20 s, and dried by filtered air flow (Figure 1c).

### 2.10. Hybridizations, Detections and Statistical Analyses

Specific fluorescent DENV probes (Table 2) were hybridised to the immobilized DNA microarray targets. A volume of 5 μL containing specific probes for each DENV serotype (1–4) was used as shown in Figure 1d. The microarray substrates and coverslips were sealed in a humidity chamber (Bio-Rad Laboratories, Hercules, CA, USA) and incubated at 65 °C for at least 6 h. The optimal concentration of the probes for DENV-1 to -4 was 20 μM. The coverslips and supports were gently removed (4× SSC in a wash bottle) and the arrays were washed by immersion in 1× SSC, 0.1% SDS for 10 min (10 slides in 250 mL), 0.1× SSC, 0.1% SDS twice for 10 min, and 0.1× SSC twice for 10 min. Finally, the slides were blown dry by pushing the liquid away from the spots and toward the outer edges. The slides were scanned using a GeneTac LS IV Scanner (Genomic Solutions) to detect Texas Red, FAM, Cy5, and Yakima yellow fluorescence at excitation line wavelengths of 583, 494, 530, and 646 nm, respectively. Figure 1e illustrates this procedure. Fluorescence values were obtained using TM4 software [40] and statistical analyses were performed in S-Plus Statistics Software [41]. Normalization of the means was performed after background correction. The microarray data have been deposited in the NCBI Gene Expression Omnibus database (http://www.ncbi.nlm.nih.gov/geo/) with GEO series accession number GPL11294-CINVESTAV-Dengue 0.6k Diagnostic Chip.

## 3. Results and Discussion

### 3.1. Selection of Probes for the Detection of DENV Serotype

To select effective primer pairs from published candidates (Table 2), we tested each PCR primer pair in a separate reaction for each serotype. Standard viral RNAs of DENV-1, strain Hawaii (HA1-1944); DENV-2, strain New Guinea C (NGC-1944); DENV-3, strain Philippines (H-87); and DENV-4, strain Philippines (H-241-1946) were assessed. Results measured by amplicon density are shown in Table 3. Optimal reproducibility was obtained with consensus primer DV1 and specific primers DSP1 to DSP4 (Table 2). DSP1–4 were labelled as described in the Experimental Section (Table 2).

### 3.2. DNA Spot Concentration and Microarray Optimisation

**Figure 2.**
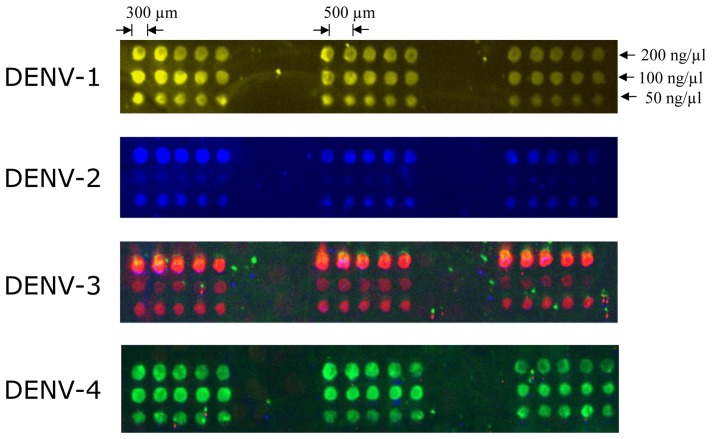
Hybridisation patterns of representative DENV serotypes, indicated on the left side of each array. Vertical lanes represent different RT-PCR product concentrations (50, 100, and 200 ng·μL^−1^, respectively) for each DENV serotype. Specific labelled primers (Texas Red for DENV-1, FAM for DENV-2, CY5 for DENV-3 and Yakima Yellow for DENV-4) were hybridised as described in the Experimental Section and the arrays were spotted in triplicate.

Target density on the array surface must be optimised for effective hybridization. Several target concentrations (ranging from 50, 100, and 200 ng·μL^−1^) were tested as shown in Figure 2. To determine the surface density, further hybridization experiments were performed with fluorochrome-labelled probes. We found that the ideal DNA target concentration for fluorescence detection with the complementary labelled probe (20 μM) was 50 ng·μL^−1^. Triplicate arrays of five spots from the same sample produced virtually identical images. Probe specificity is also shown in Figure 2.

### 3.3. Detection of Viral RNA in Clinical Serum Samples

**Figure 3.**
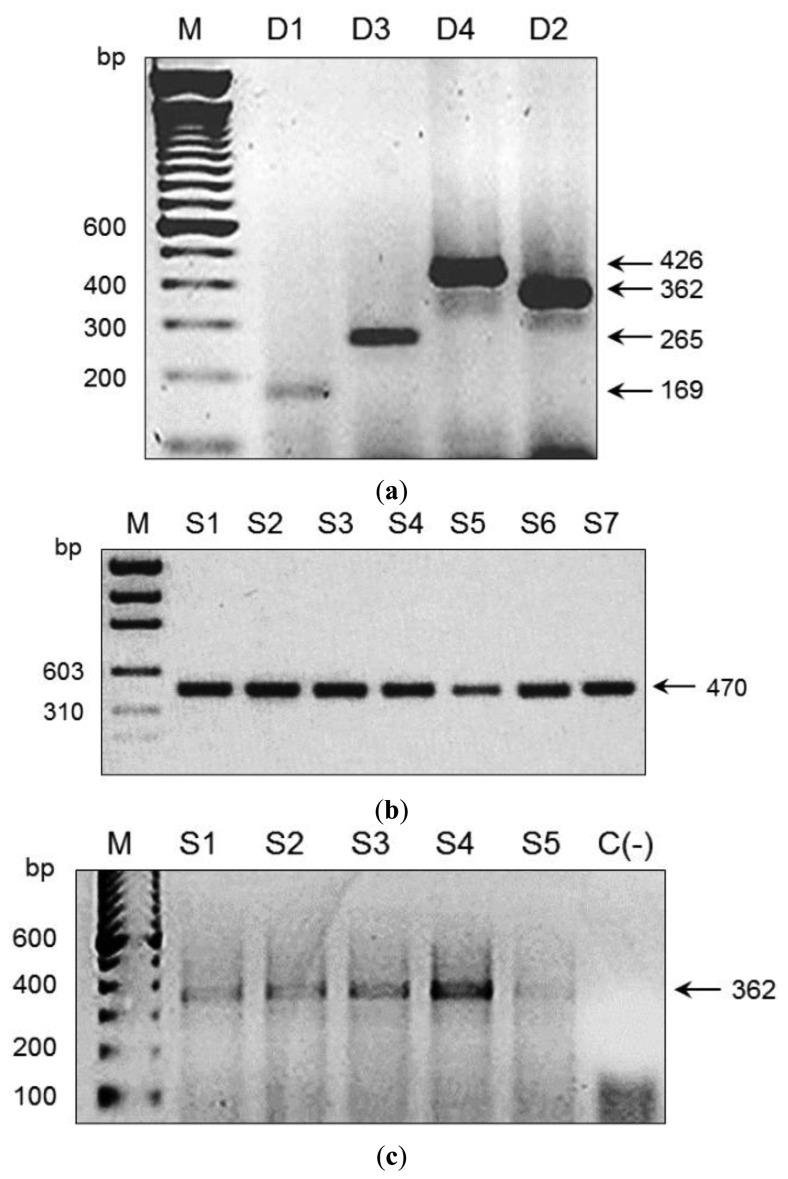
Amplification products of RT-PCR consensus and serotype-specific primer pairs. The RT-PCR products were observed in ethidium bromide-stained agarose gels (2%). Input viral RNAs were extracted from each control dengue virus strain. Results for serotypes DEN-1, DEN-2, DEN-3, and DEN-4 are shown. (**a**) The 169-, 362-, 265-, and 426-bp amplicons for DEN-1, DEN-2, DEN-3, and DEN-4, respectively. (**b**) The 470-bp amplicons expected for all four dengue serotypes using the consensus primers. (**c**) The 362-bp amplicons for DEN-2 obtained from five samples.

**Figure 4.**
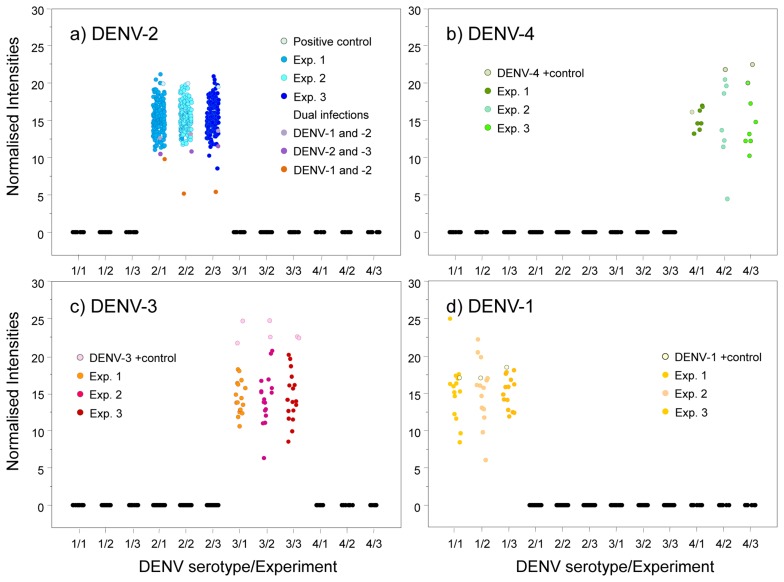
Samples were evaluated for the presence of DENV-1, -2, -3, and -4. Plots represent the normalised intensity for each sample in three different experiments. Each panel represents values for DENV-1 (Texas Red), DENV-2 (FAM), DENV-3 (Cy5), or DENV-4 (Yakima yellow). (**a**) Fluorescence for 162 DENV-2-positive samples, 1 positive control, and 3 dual infections positive for DENV-1 and -2, DENV-2 and -3, and DENV-1 and -2. (**b**) Seven DENV-4-positive samples and 1 positive control. (**c**) Seventeen DENV-3-positive samples and 2 positive controls. (**d**) Fourteen DENV-1-positive samples and 1 positive control. All positive samples yielded fluorescence intensities >0.2. Each experiment is represented by spots of different colours. Each spot represents the average fluorescence intensity of triplicate spots. Analysis was performed in S-Plus software [S-PLUS: Copyright 1988, 2007 Insightful Corp. Rev Date:Wed May 02 10:07:00 2007 Buil8052. Seria#: WTB00000001. Juan Burgueño. CIMMYT, INC; 41].

We used an amplicon derived from two consensus primers (DV1 and DV3) which contain the most conserved and specific sequences for each serotype, thus maximising the probability that all members of each viral serotype could be detected; this is a difficult and problematic task when using traditional methods. To assess the performance of our microarray in a clinical setting, the presence of DENV was evaluated in 197 human serum specimens from 12 Oaxaca cities and Veracruz. Results were validated by visual detection of the amplicon obtained with DV1 and specific primers DSP1-4 in agarose gels (Figure 3); this Figure also shows amplicons produced by consensus primers DV1 and DV3. Microarray results revealed DENV-2 in 88 samples from Oaxaca and 41 from Veracruz; DENV-3 in two samples from Oaxaca and ten from Veracruz; DENV-1 in six samples from Veracruz; and DENV-4 in seven samples from Veracruz (Figure 4). Dual infections with DENV-2 and -3 in one sample from Oaxaca and DENV-1 and -2 in a sample from Veracruz were also detected (Figure 4a). DENV serotype identity was confirmed in all samples by determining amplicon size (Figure 3). Microarray results are shown in Figure 4; two samples from the 197 analysed serum specimens that were reported as negative for DENV were also negative in this assay. Thirty-seven symptomatic patients were negative, and eleven that were reported as negative by the Mac-ELISA (IgM Antibody Capture Enzyme Linked Immunosorbent Assay) were positive by microarray analysis and confirmed by 2% agarose gel electrophoresis of the RT-PCR products. Figure 3 shows an example of these amplifications. Each assay included negative and positive controls, as well as RT-PCR products of known strains maintained in culture (isolates, VER1.1.5 for DENV-1; VER3.5.1 for DENV-2; VER1.2.1 for DENV-3, and VER1.2.7 for DENV-4). All negative controls were confirmed as negative by the microarray analysis. There were no false-negative samples, indicating the high specificity of this method. The microarray image platforms were submitted to GEO NCBI (Accession number: GPL11294, CINVESTAV, Dengue 0.6k Diagnostic Chip). These results confirmed the circulation of the four serotypes in the states of Oaxaca and Veracruz, Mexico (36). This study also showed that DENV-2 is present in serum at a higher frequency, consistent with previous assessments of the prevalence of these serotypes in the epidemics of 2000–2001 [37] and 2005–2006 [20,21].

### 3.4. Detection of Viral RNA in Field-Collected Mosquito Vectors of Dengue

Microarrays were also used to monitor and type DENV RNA in *Aedes* mosquitoes caught in the field (Table 1). Mosquito collection was conducted in 76 cities and 19 states with reported dengue cases (Table 1). DENV was detected in 35 cities: 28 contained DENV-2, six contained DENV-1, and three contained DENV-3 (Table 4). Mosquito pools collected in the states of Quintana Roo, Puebla, Hidalgo, Mexico, Queretaro, Guanajuato, Michoacán, and Jalisco did not contain DENV RNA. Table 1 shows the number of pools collected per city and state; Table 4 and Figure 4 illustrate serotype detection by microarray analysis. Figure 3 also shows representative examples of validation by agarose gel electrophoresis of RT-PCR products using the consensus primer DV1 and specific primers DSP1-4. All assays included positive controls for each serotype maintained in cell culture (isolates VER1.1.5 for DENV-1; VER3.5.1 for DENV-2; VER1.2.1 for DENV-3, and VER1.2.7 for DENV-4) and negative controls (42 spots) with RT-PCR products of non-infected cultured mosquito cells (Accession number: GPL11294, CINVESTAV, Dengue 0.6k Diagnostic Chip).

These results are crucial because these advances in microarray technology have made it possible to detect the dengue viral nucleic acid in mosquito pools from the field and in human serum specimens. This procedure was sensitive and specific for the detection of DENV up to the serotype level and dual infections (Figure 4, Table 4). Viral RNA in two of the mosquito pools (Yautepec, Morelos and Tepic, Nayarit) was not detected by RT-PCR and gel electrophoresis (Figure 3), but DENV-1 was detected in these samples by microarray analysis. This suggests that the sensitivity of the microarray may be higher than that of gel electrophoresis. We were able to detect the specific amplicon for DENV-1 by agarose gel electrophoresis using a higher concentration of cDNA. Microarray analysis also showed DENV-2 in seven pools of *Ae. albopictus* from Tapachula (Chiapas) and 11 pools of *Ae. aegypti* (Table 2); eleven pools of *Ae. aegypti* from Tavela (Oaxaca) contained DENV-2 and 3 DENV-3; of 30 mosquito pools of *Ae. aegypti* collected in Ciudad Mante (Tamaulipas), 21 contained DENV-2, six contained DENV-1, and three contained DENV-1 and -2 (Table 2).

**Figure 5.**
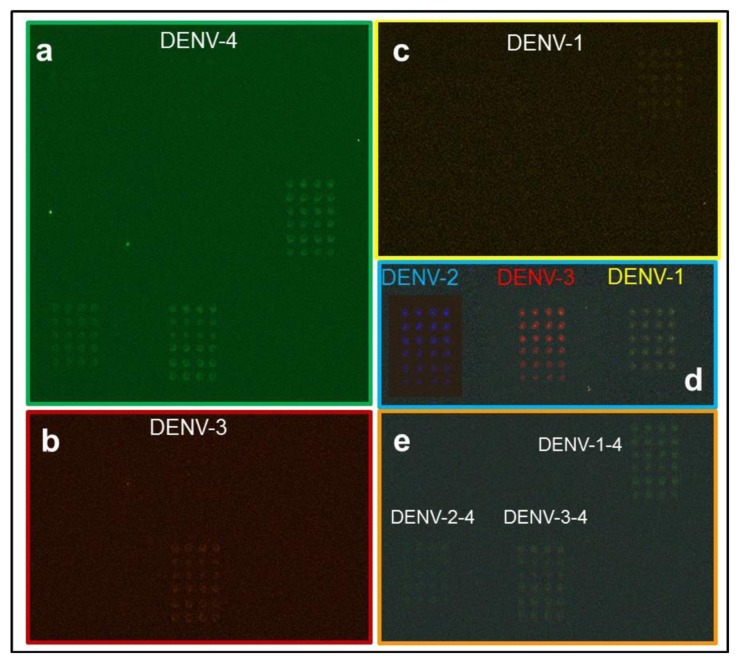
Samples were evaluated for the presence of dual serotypes. Panels (**a**)–(**c**) represent fluorescence for DENV-4, -3, and -1; (**d**) corresponds to DENV-1 (Texas Red), DENV-2 (FAM), DENV-3 (Cy5) and DENV-4 (Yakima yellow) serotype controls; and (**e**) shows the merged images of DENV-1 and -4, DENV-2 and -4, and DENV-3 and -4.

Serum and mosquito samples revealed the presence of co-incident serotypes DENV-1 and -2 and DENV-2 and -3. To test other serotype combinations, amplicons of DENV-1 and -4, DENV-2 and -4, and DENV-3 and -4 were combined and tested in the microarray assay. The results support the ability of this system to detect double infections with different serotypes (Figure 5).

Although the level of detection was apparently equivalent to RT-PCR-based detection alone, microarrays have the advantage of high throughput and high sensitivity for low levels of cDNA. The detection limit of an amplicon on a standard agarose gel is approximately 20–30 ng; in the microarray, this limit is 0.05–0.2 ng cDNA per spot, derived from 1 nl of a 50–200 ng·μL^−1^ solution.

We also detected DENV serotype 2 in mosquito pools of *Ae. albopictus*, suggesting this species probably serves as a maintenance vector in rural areas of dengue-endemic countries such as Southeast Asia and the Pacific islands. This finding is also very important because this mosquito vector also has the potential to transmit DENV.

### 3.5. Statistical Analysis

Probe specificity was analysed by normalising the fluorescence intensities for all fluorochromes. Our results showed that only one sample yielded reproducible fluorescence signals for DENV-1 and DENV-2 (Figure 4a, yellow spots). Thus, the probes have a high specificity to diagnose the four DENV serotypes even when two different serotypes are present in the same sample.

After analysing the variability of the fluorescence-normalised values, we found that the variability between samples was higher than the variability between triplicate samples, with the exception of the Yakima yellow results in experiment 2 and the Cy5 results in experiment 1 (Table 5).

The primary advantages of this microarray technique include the speed at which high numbers of specimens can be screened for the presence of DENV with high sensitivity and specificity for single and dual serotype infections. The speed of specimen detection can be improved by using a microfluidic microarray system [42,43]. Fluorescence variability was very low in our assays, suggesting this technology will be very useful for DENV diagnoses. Detection of two cases of dual infections (DENV-2 and -1; and DENV-2 and -3) confirm the feasibility of using this microarray assay to diagnose dual infections (Figure 4) [20,21,44].

This and previous reports [45–47] have demonstrated the great potential of microarray technology for viral detection and identification. These studies pave the way to designing a universal viral signature chip that can be used to determine the presence of DENV, as well as the specific serotype, in a given sample. The experience gained in this study can be applied to any method that relies on the formation of hybrids between sample-derived nucleic acids and short oligonucleotide probes. Additional work is needed to test *in silico*-designed primers for detecting additional DENV variants on the same chip, thereby supporting studies of DENV evolution across different geographic areas.

We have developed a chip that may be used for diagnosis for a large number of human serum specimens or specimens from mosquitoes and have demonstrated its utility for the identification and surveillance of DENV serotypes. The chip can be used to detect other viruses, since the target amplicon was obtained with consensus primers (DV1 and DV3) that recognize other flaviviruses such as Japanese encephalitis, Kunjin, and yellow fever. This level of sensitivity is very important because these viruses induce symptoms clinically indistinguishable from those of true DENV. We plan to develop such a chip with primers specific to these additional flaviviruses.

Robotic microarray techniques and laser-based image analysis have been applied to the design and development of DNA microarrays to analyse transcriptional responses of cells and microorganisms [33,43]. The use of defined oligonucleotide probes is especially attractive for the synthesis of specific microarrays for viral pathogens circulating in large human populations [47]. The first reported chip assay for a human pathogen (human herpes virus) was highly effective in a global assessment of human cytomegalovirus [48] and for more recent evaluations of genetic variability in West Nile virus [32]. We used a similar approach to synthesise a chip specifically for DENV serotype determination. The high specificity of the probes described in this study can also be adapted to the recently described DNA Biosensor base on a nanoporous alumina membrane [49] while maintaining specificity for each DENV serotype.

## 4. Conclusions/Outlook

We have developed a microarray for accurate typing of DENV serotypes in human serum or mosquitoes; the system can distinguish serotypes 1, 2, 3, and 4 as well as dual infections with different serotypes. This microarray may also detect other human animal flaviviruses, since the consensus primers DV1 and DV3 target RNA from Japanese encephalitis, Kunjin, and yellow fever viruses. This diagnostic tool has many applications in the study of viral pathogenesis and, perhaps equally importantly, may facilitate viral identification and infections caused by two different DENV serotypes.

## Figures and Tables

**Table 1.** Locations, dates of collections, global positioning system coordinates and sample sizes of *Ae. aegypti* collections in Mexico.

**State**	**City**	**Collection Date**	**Coordinates (Latitude/Longitude)**	**Female Pools**
	Cancun	07/08/2005	21°09′00.93″/86°49′39.20″	19
Quintana Roo	Cozumel	08/08/2005	20°30′31.53″/86°56′45.42″	6
	Felipe Carrillo Puerto	09/08/2005	19°35′12.24″/88°02′22.67″	11
	Puerto Arturo	11/08/2005	19°39′51.45″/89°04′57.21″	13
	La Esperanza	13/08/2005	21°02′09.70″/87°38′33.46″	14
Yucatan	Tinum	15/08/2005	20°46′03.22″/88°23′18.78″	6
	Ticum	17/08/2005	20°08′50.65″/89°12′54.87″	11
	Merida	19/08/2005	20°59′31.05″/89°38′08.56″	19
	Hopelchen	21/08/2005	19°44′37.80″/89°50′38.49″	9
Campeche	Calakmul	23/08/2005	18°21′18.04″/89°32′15.35″	19
	Francisco Escarcega	25/08/2005	18°36′00.02″/90°43′41.11″	13
	Palizada	27/08/2005	18°15′15.04″/92°05′25.22″	8
	Tenosique	04/09/2005	17°28′54.38″/91°24′59.10″	19
Tabasco	Jalapa	06/09/2005	17°43′25.45″/92°48′44.87″	11
	Villahermosa	08/09/2005	17°59′19.10″/92°55′54.18″	23
	Paraiso	10/09/2005	18°24′14.18″/93°12′17.94″	17
	Ococingo	12/09/2005	16°53′54.06″/92°06′14.24″	21
Chiapas	Tapachula	14/09/2005	14°52′03.61″/92°16′18.06″	21
	Tuxtla Gutierrez	16/09/2005	16°45′48.19″/93°06′09.98″	9
	Arriaga	18/09/2005	16°14′24.01″/93°54′34.13″	14
	Acayucan	02/10/2005	17°57′92.90″/94°55′15.11″	39
Veracruz	Tierra Blanca	04/10/2005	18°27′33.10″/96°21′24.48″	18
	Xalapa	06/10/2005	19°33′12.88″/96°54′44.00″	11
	Tantoyuca	08/10/2005	21°21′10.21″/98°13′08.71″	22
	Tavela	10/10/2005	16°39′37.79″/95°59′58.37″	14
	Pochutla	12/10/2005	15°44′52.55″/96°28′09.64″	37
	Oaxaca I	14/07/2004	17°02′34.13″/96°43′06.91″	22
Oaxaca	Oaxaca II	14/10/2005	17°02′34.13″/96°43′06.91″	17
	Tuxtepec I	17/07/2003	18°04′93.70″/96°06′78.30″	43
	Tuxtepec II	02/09/2004	18°04′93.70″/96°06′78.30″	45
	Tuxtepec III	16/10/2005	18°04′93.70″/96°06′78.30″	11
	Nanahuatipan	19/10/2005	18°07′21.42″/97°06′45.81″	17
	Venustiano Carranza	16/07/2005	20°30′31.30″/97°40′16.54″	11
	Acateno	17/07/2005	20°07′59.74″/97°12′18.12″	9
Puebla	Acatlan	18/07/2005	18°12′00.94″/98°02′53.64″	2
	Izucar de Matamoros	19/07/2005	18°35′52.52″/98°28′28.68″	13
	Ometepec	21/10/2005	16°41′04.02″/98°24′03.72″	4
Guerrero	Huamuxtitlan	22/10/2005	17°47′50.38″/98°33′47.22″	22
	Huitzuco	23/10/2005	18°18′31.09″/99°20′37.98″	12
	Petatlan	24/10/2005	17°32′12.48″/101°16′09.65″	17
	Tepalcingo	31/05/2005	18°35′57.97″/98°50′25.78″	8
Morelos	Yautepec	31/05/2005	18°52′52.76″/99°04′02.78″	14
	Xochitepec	30/05/2005	18°48′07.18″/99°14′08.09″	11
	Puente de Ixtla	30/05/2005	18°36′43.83″/99°19′58.28″	9
	Huehuetla	27/06/2005	20°31′37.64″/98°01′15.78″	4
Hidalgo	Jaltocan	28/06/2005	21°10′23.65″/98°35′58.86″	2
	Metzitlan	29/06/2005	20°35′31.25″/98°45′43.53″	6
	Tlanalapa	30/06/2005	19°49′38.55″/98°36′15.79″	3
	Malinalco	02/07/2005	18°46′49.23″/99°32′59.21″	14
Mexico	Nuevo Santo Tomas	03/07/2005	19°10′26.34″/100°17′18.95″	7
	Tejupilco	04/07/2005	18°53′23.32″/100°08′25.64″	9
	Tlatlaya	05/07/2005	18°32′17.37″/100°13′47.38″	9
	Matamoros	24/07/2005	25°50′36.99″/97°27′45.08″	8
Tamaulipas	Soto la Marina	25/07/2005	23°46′09.07″/98°12′44.45″	9
	Ciudad Mante	27/07/2005	22°45′14.20″/98°59′28.66″	31
	Nuevo Laredo	29/07/2005	27°29′06.75″/99°31′47.13″	3
	Tancoyol	04/06/2005	21°28′38.70″/99°18′53.55″	4
Queretaro	Landa de Matamoros	05/06/2005	21°11′12.42″/99°19′13.32″	8
	Jalpan	06/06/2005	21°10′17.79″/99°25′14.23″	3
	Arroyo Seco	07/06/2005	21°32′50.05″/99°41′15.58″	7
	Guanajuato	09/06/2005	21°01′12.33″/101°15′56.92″	7
Guanajuato	Irapuato	11/06/2005	20°42′08.61″/101°22′12.88″	13
	Abasolo	13/06/2005	20°27′04.59″/101°32′04.86″	2
	Penjamo	15/06/2005	20°26′13.61″/101°43′03.84″	6
	Huetamo	19/06/2005	18°37′11.89″/100°53′25.31″	14
Michoacan	Tacambaro	21/06/2005	19°14′29.25″/101°27′13.96″	7
	Lázaro Cardenas	23/06/2005	17°58′21.82″/102°13′41.12″	5
	Apatzingan	25/06/2005	19°05′21.86″/102°22′08.41″	9
	Colima	26/09/2005	19°14′43.90″/103°42′23.33″	21
Colima	Tecoman	27/09/2005	18°54′54.16″/103°53′12.38″	9
	Minatitlan	28/09/2005	19°23′16.59″/104°03′17.13″	4
	Manzanillo	29/09/2005	19°03′35.47″/104°17′18.53″	8
	Pihuamo	22/05/2005	19°15′24.83″/103°22′33.15″	9
Jalisco	Tequila	24/05/2005	20°53′20.25″/103°50′02.26″	21
	Cihuatlan	26/05/2005	19°13′58.41″/104°34′17.31″	4
	Mismaloya	28/05/2005	20°31′52.79″/105°17′09.14″	6
	Ixtlan del Rio	14/05/2005	21°01′52.83″/104°22′30.69″	8
Nayarit	Tepic	16/05/2005	21°30′04.94″/104°53′04.03″	16
	Ixcuintla	18/05/2005	21°48′04.57″/105°12′13.70″	6
	Acaponeta	20/05/2005	22°29′23.95″/105°22′29.68″	4

*Aedes aegypti* were collected in all cities;

* Pools of *Aedes albopictus* collected.

**Table 2.** Probes and primers used for the detection of DENV serotype.

**Primer Name**	**Sequence**	**Genome Postition**	**Gen**	**Amplicon Size**
Probes				
Texas RedDENV-1	5′-(Texas Red) AGTTTCTTTTCCTAAACACCTCG-3′	5045–5067	NS3	
6FAMDENV-2	5′-(_6 FAM) CCGGTGTGCTCRGCYCTGAT-3′	5260–5279	NS3	
CY5DENV-3	5′-(_CY5) TTAGAGTYCTTAAGCGTCTCTTG-3′	5152–5174	NS3	
YakimaYellowDENV-4	5′-(_Yakima Yellow) CCTGGTTGATGACAAAAGTCTTG-3′	5320–5342	NS3	
Primers				
AD3 (-) [14]	5′-CTGATTTCCAT(A, C,G,T)CC(A,G)TA-3′	3412–3428	NS1	
AD4 (+)	5′-GA(C,T)ATGGG(C,G,T)TA(C,T)TGGATAGA-3′	3010–3029	NS1	419(AD3 and AD4)
D1	5′-GAGGACCAATATCTCAG-3′	3135–3151	NS1	265 (D1 and AD3)
D2 (+)	5′-AAGCTTGAGATGGACTTT-3′	3235–3151	NS1	194 (D2 and AD3)
D3 (+)	5′-GTCTAGCTGGTCCCATT-3′	3150–3166	NS1	271 (D3 and AD3)
D4 (+)	5′-ATCATATGCGGGCCCTT-3′	3155–3171	NS1	273 (D4 and AD3)
D1 [15]	5′-TCAATATGCTGAAACGCGCGAGAAACCG-3′	134–161	C-prM	
D2	5′-TTGCACCAACAGTCAATGTCTTCAGGTTC-3′	616–644	C-prM	511 (D1 and D2)
TS1	5′-CGTCTCAGTGATCCGGGGG-3′	568–586	C-prM	482 (Dl and TS1)
TS2	5′-CGCCACAAGGGCCATGAACAG-3′	232–252	C-prM	119 (Dl and TS2)
TS3	5′-TAACATCATCATGAGACAGAGC-3′	400–421	C-prM	290 (Dl and TS3)
TS4	5′-CTCTGTTGTCTTAAACAAGAGA-3′	506–527	C-prM	392 (Dl and TS4)
Bio-CFDJ9977a [12]	5′- GCATGTCTTCCGTCGTCATCC-3′	9952–9977	NS5	
DEN1-J9243	5′-GCCTGAACATGCTCTATTGGCT-3′	9243–9264	NS5	761 (J9243 and CFDJ)
DEN2-J9452	5′-TCTTCAAAAGCATTCAGCACCT-3′	9452–9473	NS5	546 (J9452 and CFDJ)
DEN1-J9243	5′-GCCTGAACATGCTCTATTGGCT-3′	9243–9264	NS5	761 (J9243 and CFDJ)
DEN2-J9452	5′-TCTTCAAAAGCATTCAGCACCT-3′	9452–9473	NS5	546 (J9452 and CFDJ)
DEN3-9471	5′-CCCATCCGCTAGAGAAGAAAATTACAC-3′	9471–9497	NS5	522 (J9471 and CFDJ)
DEN4-9580	5′- GGTTTGGCACTTCCCTCCTCTTCTTG-3′	9580–9605	NS5	411 (9580 and CFDJ)
DV1 (+) [30]	5′-GGRACKTCAGGWTCTCC-3′	4918–4934	NS3	
DV3	5′-AARTGIGCYTCRTCCAT-3′	5368–5384	NS3	470 (DV1 and DV3)
DSP1 (-) [16]	5′-AGTTTCTTTTCCTAAACACCTCG-3′	5067–5045	NS3	169 (DV1 and DSP1)
DSP2 (-)	5′-CCGGTGTGCTCRGCYCTGAT-3′	5279–5260	NS3	362 (DV1 and DSP2
DSP3 (-)	5′-TTAGAGTYCTTAAGCGTCTCTTG-3′	5174–5152	NS3	265 (DV1 and DSP3
DSP4 (-)	5′-CCTGGTTGATGACAAAAGTCTTG-3′	5342–5320	NS3	426 (DV1 and DSP4)

**Table 3.** Amplicon quantification by densitometry.

**Serotype**	**Primers** *	**Amount of DNA (ng)**
DENV-1	D1 and AD3	128
	D1 and TS3	89
	DEN1-J9243 and CFDJ	45
	DV1 and DSP1	169
DENV-2	D2 and AD3	160
	Dl and TS2	114
	DEN2-J9452 and CFDJ	39
	DV1 and DSP2	199
DENV-3	D3 and AD3	33
	Dl and TS3	92
	DEN3-J9471 and CFDJ	41
	DV1 and DSP3	156
DENV-4	D4 and AD3	25
	Dl and TS4	24
	DEN4-9580 and CFDJ	30
	DV1 and DSP4	187

* Table 2 lists primer sequences.

**Table 4.** Mosquito pools tested for DENV by microarray technology and validation by RT-PCR and agarose gel electrophoresis.

**State**	**City**	**Infected Mosquito Pools**	**DENV Serotype** *	

			**Microarray**	**RT-PCR**
Yucatan	La Esperanza	7	2	2
	Tinum	5	1	1
	Ticum	10	2	2
	Mérida	5	2	2
Campeche	Hopelchen	9	2	2
	Calakmul	11	2	2
	Francisco Escarcega	3	2	2
	Palizada	7	2	2
Tabasco	Tenosique	14	3	3
	Jalapa	4	2	2
	Villahermosa	1	2	2
	Paraíso	9	1	1
Chiapas	Ococingo	2	2	2
	Tapachula **	7 *	2	2
	Tapachula	11	2	2
	Tuxtla Gutierrez	8	2	2
Veracruz	Acayucan	6	3	3
	Tierra Blanca	7	2	2
	Xalapa	11	2	2
	Tantoyuca	2	2	2
Oaxaca	Tavela	11	2	2
		2	3	3
		1	3	3
	Pochutla	4	2	2
	Oaxaca (I, II)	10	2	2
	Tuxtepec (I, II, III)	31	2	2
	Nanahuatipan	12	2	2
Guerrero	Huamuxtitlan	17	2	2
	Petatlan	2	2	2
Morelos	Yautepec	1	1	ND
Tamaulipas	Matamoros	8	2	2
	Soto la Marina	9	2	2
	Ciudad Mante	21	2	2
		6	1	1
		3	1,2	1,2
	Nuevo Laredo	3	2	2
Colima	Colima	21	2	2
	Tecoman	9	1	1
	Minatitlan	4	2	2
Nayarit	Tepic	1	1	ND

* DENV serotype was determine by the microarray technology and validated by the size differences between amplicons obtained after amplification by specific primer;

** *Ae. Albopictus* pools; ND. Not detected.

**Table 5.** Variability coefficient (%) of sample fluorescence in the microarray.

**Label**	**Samples** *	**Experiment**		

		**1**	**2**	**3**
		Fluorescence-Normalised Value (%)		
FAM	DENV-2	**13.5**	**12.6**	**14.5**
		7.4	7.5	5.0
Yellow	DENV-4	**9.2**	**33.2**	**27.1**
		3.3	33.6	10.6
	DENV-3	**19.4**	**26.1**	**24.3**
		21.3	20.9	16.7
	DENV-1	**25.1**	**25.8**	**12.8**
		8.8	15.8	12.7

Variability between analysed samples (bold font); Variability between replicates (normal font).
